# Early Identification of Autism Spectrum Disorder Among Children Aged 4 Years — Autism and Developmental Disabilities Monitoring Network, 11 Sites, United States, 2018

**DOI:** 10.15585/mmwr.ss7010a1

**Published:** 2021-12-03

**Authors:** Kelly A. Shaw, Matthew J. Maenner, Amanda V. Bakian, Deborah A. Bilder, Maureen S. Durkin, Sarah M. Furnier, Michelle M. Hughes, Mary Patrick, Karen Pierce, Angelica Salinas, Josephine Shenouda, Alison Vehorn, Zachary Warren, Walter Zahorodny, John N. Constantino, Monica DiRienzo, Amy Esler, Robert T. Fitzgerald, Andrea Grzybowski, Allison Hudson, Margaret H. Spivey, Akilah Ali, Jennifer G. Andrews, Thaer Baroud, Johanna Gutierrez, Libby Hallas, Jennifer Hall-Lande, Amy Hewitt, Li-Ching Lee, Maya Lopez, Kristen Clancy Mancilla, Dedria McArthur, Sydney Pettygrove, Jenny N. Poynter, Yvette D. Schwenk, Anita Washington, Susan Williams, Mary E. Cogswell

**Affiliations:** ^1^National Center on Birth Defects and Developmental Disabilities, CDC, Atlanta, Georgia; ^2^University of Utah School of Medicine, Salt Lake City, Utah; ^3^University of Wisconsin, Madison, Wisconsin; ^4^University of California, San Diego, California; ^5^Rutgers New Jersey Medical School, Newark, New Jersey; ^6^Vanderbilt University Medical Center, Nashville, Tennessee; ^7^Washington University, St. Louis, Missouri; ^8^University of Minnesota, Minneapolis, Minnesota; ^9^University of Arkansas for Medical Sciences, Little Rock, Arkansas; ^10^Johns Hopkins University, Baltimore, Maryland; ^11^Oak Ridge Institute for Science and Education, Oak Ridge, Tennessee; ^12^University of Arizona, Tucson, Arizona

## Abstract

**Problem/Condition:**

Autism spectrum disorder (ASD).

**Period Covered:**

2018.

**Description of System:**

The Autism and Developmental Disabilities Monitoring Network is an active surveillance program that estimates ASD prevalence and monitors timing of ASD identification among children aged 4 and 8 years. This report focuses on children aged 4 years in 2018, who were born in 2014 and had a parent or guardian who lived in the surveillance area in one of 11 sites (Arizona, Arkansas, California, Georgia, Maryland, Minnesota, Missouri, New Jersey, Tennessee, Utah, and Wisconsin) at any time during 2018. Children were classified as having ASD if they ever received 1) an ASD diagnostic statement (diagnosis) in an evaluation, 2) a special education classification of ASD (eligibility), or 3) an ASD International Classification of Diseases (ICD) code. Suspected ASD also was tracked among children aged 4 years. Children who did not meet the case definition for ASD were classified as having suspected ASD if their records contained a qualified professional’s statement indicating a suspicion of ASD.

**Results:**

For 2018, the overall ASD prevalence was 17.0 per 1,000 (one in 59) children aged 4 years. Prevalence varied from 9.1 per 1,000 in Utah to 41.6 per 1,000 in California. At every site, prevalence was higher among boys than girls, with an overall male-to-female prevalence ratio of 3.4. Prevalence of ASD among children aged 4 years was lower among non-Hispanic White (White) children (12.9 per 1,000) than among non-Hispanic Black (Black) children (16.6 per 1,000), Hispanic children (21.1 per 1,000), and Asian/Pacific Islander (A/PI) children (22.7 per 1,000). Among children aged 4 years with ASD and information on intellectual ability, 52% met the surveillance case definition of co-occurring intellectual disability (intelligence quotient ≤70 or an examiner’s statement of intellectual disability documented in an evaluation). Of children aged 4 years with ASD, 72% had a first evaluation at age ≤36 months. Stratified by census-tract–level median household income (MHI) tertile, a lower percentage of children with ASD and intellectual disability was evaluated by age 36 months in the low MHI tertile (72%) than in the high MHI tertile (84%). Cumulative incidence of ASD diagnosis or eligibility received by age 48 months was 1.5 times as high among children aged 4 years (13.6 per 1,000 children born in 2014) as among those aged 8 years (8.9 per 1,000 children born in 2010). Across MHI tertiles, higher cumulative incidence of ASD diagnosis or eligibility received by age 48 months was associated with lower MHI. Suspected ASD prevalence was 2.6 per 1,000 children aged 4 years, meaning for every six children with ASD, one child had suspected ASD. The combined prevalence of ASD and suspected ASD (19.7 per 1,000 children aged 4 years) was lower than ASD prevalence among children aged 8 years (23.0 per 1,000 children aged 8 years).

**Interpretation:**

Groups with historically lower prevalence of ASD (non-White and lower MHI) had higher prevalence and cumulative incidence of ASD among children aged 4 years in 2018, suggesting progress in identification among these groups. However, a lower percentage of children with ASD and intellectual disability in the low MHI tertile were evaluated by age 36 months than in the high MHI group, indicating disparity in timely evaluation. Children aged 4 years had a higher cumulative incidence of diagnosis or eligibility by age 48 months compared with children aged 8 years, indicating improvement in early identification of ASD. The overall prevalence for children aged 4 years was less than children aged 8 years, even when prevalence of children suspected of having ASD by age 4 years is included. This finding suggests that many children identified after age 4 years do not have suspected ASD documented by age 48 months.

**Public Health Action:**

Children born in 2014 were more likely to be identified with ASD by age 48 months than children born in 2010, indicating increased early identification. However, ASD identification among children aged 4 years varied by site, suggesting opportunities to examine developmental screening and diagnostic practices that promote earlier identification. Children aged 4 years also were more likely to have co-occurring intellectual disability than children aged 8 years, suggesting that improvement in the early identification and evaluation of developmental concerns outside of cognitive impairments is still needed. Improving early identification of ASD could lead to earlier receipt of evidence-based interventions and potentially improve developmental outcomes.

## Introduction

Autism spectrum disorder (ASD) is a developmental disability characterized by deficits in social communication and interaction and the presence of restricted interests and repetitive behaviors. Early routine screening for ASD and other developmental concerns is recommended by the American Academy of Pediatrics (*1*) because early evaluation, diagnosis, and evidence-based interventions could enhance short-term and long-term developmental outcomes for young children with ASD (*2*–*6*). Because of the potential to improve outcomes, increasing the proportion of all children who receive a developmental screening by age 36 months and the proportion of children with ASD who receive special services by age 48 months are included as Healthy People 2030 goals (*7*).

Since 2010, CDC has conducted active population-based surveillance of ASD among children aged 4 years as a subset of the Autism and Developmental Disabilities Monitoring (ADDM) Network to better understand early identification of ASD in communities. Surveillance among children aged 4 years in 2016 (*8*) indicated similar prevalence among children of different racial and ethnic groups, consistent with trends among children aged 8 years (*9*). In addition, ASD identification measured by cumulative incidence of diagnosis by age 48 months was higher among children aged 4 years (born in 2012) compared with children aged 8 years (born in 2008), suggesting increased early identification of ASD among the younger cohort (*8*). Cumulative incidence represents the number of children identified at or before each month of age divided by the total population and, unlike median age calculations, it reflects differences in prevalence and allows direct age-by-age comparison over time and between groups of children (*9*,*10*).

For surveillance year 2018, ASD surveillance among children aged 4 years expanded from a subset to all ADDM Network sites. For this age group, sites ascertained children with characteristics meeting the ASD case definition as well as those who were suspected of having ASD by a qualified provider.

This report focuses on early identification of children with ASD and presents the estimated prevalence of ASD and suspected ASD among children aged 4 years, cumulative incidence of ASD identified by age 48 months, and characteristics of children aged 4 years with ASD and suspected ASD identified by ADDM Network sites in 2018. These data can be used to track trends and support efforts to ensure children with ASD are identified and receive necessary evidence-based interventions as early as possible.

## Methods

### Surveillance Sites and Procedures

For 2018, all ADDM Network sites (Arizona, Arkansas, California, Georgia, Maryland, Minnesota, Missouri, New Jersey, Tennessee, Utah, and Wisconsin) conducted ASD surveillance among children aged 4 years (Table 1). Comparisons were made with the group of children aged 8 years for the same 11 sites (*11*). All sites functioned as public health authorities under the Health Insurance Portability and Accountability Act of 1996 Privacy Rule and met applicable local institutional review board, privacy, and confidentiality requirements under 45 CFR 46.

**TABLE 1:** Prevalence of autism spectrum disorder per 1,000 children aged 4 years and percentage of children who had an autism spectrum disorder diagnosis, special education eligibility, or an International Classification of Diseases code — Autism and Developmental Disabilities Monitoring Network, 11 sites, 2018

Site	Surveillance area description	Denominator	No. of children aged 4 yrs with ASD	ASD prevalence (95% CI)^*^	% of children who had an ASD diagnosis	% of children who had ASD special education eligibility	% of children who had an ASD ICD code
Arizona	Part of one county in metropolitan Phoenix	13,929^†^	141	10.1 (8.6–11.9)	92.9	24.1	44.0
Arkansas	21 counties in central Arkansas	15,387	183	11.9 (10.3–13.7)	92.3	17.5	79.2
California	Part of one county in metropolitan San Diego	16,796^†^	698	41.6 (38.6–44.7)	81.2	88.3	54.7
Georgia	Two counties in metropolitan Atlanta	23,040	340	14.8 (13.3–16.4)	75.0	48.2	60.0
Maryland	Five counties in suburban Baltimore	19,818	233	11.8 (10.3–13.4)	70.4	51.9	73.4
Minnesota	Parts of three counties in the Twin Cities metropolitan area	10,529^†^	240	22.8 (20.1–25.8)	48.3	80.4	47.5
Missouri	Five counties in metropolitan St. Louis	24,521	338	13.8 (12.4–15.3)	95.6	3.3	97.6
New Jersey	Part of two counties in New York metropolitan area	17,286^†^	342	19.8 (17.8–22.0)	98.0	17.8	73.7
Tennessee	11 counties in middle Tennessee	25,335	497	19.6 (18.0–21.4)	75.1	39.0	89.7
Utah	Three counties in northern Utah	25,064	229	9.1 (8.0–10.4)	76.9	24.0	87.8
Wisconsin	Eight counties in southeastern Wisconsin	28,689	513	17.9 (16.4–19.5)	80.3	25.5	77.2
**Total**		**220,394**	**3,754**	**17.0 (16.5–17.6)**	**80.5**	**42.9**	**72.0**

**Abbreviations:** ASD = autism spectrum disorder; CI = confidence interval; ICD = International Classification of Diseases.

* 95% CIs were calculated using the Wilson score method.

^†^ Denominator excludes school districts that were not included in the surveillance area, calculated from National Center for Education Statistics enrollment counts of kindergarteners during the 2018–19 school year.

### Case Ascertainment and Surveillance Case Definition

To identify children with ASD, health records with *International Classification of Diseases, Ninth Revision* (ICD-9) or *International Classification of Diseases, Tenth Revision* (ICD-10) billing codes relevant to developmental disabilities were requested from clinical sources, and special education records with specific special education exceptionality codes were requested from school sources. All sites had access to health records, and nine of 11 sites had access to education records covering their surveillance area (Wisconsin had partial access, and Missouri had none). Certain sites also had access to data from Individuals with Disabilities Education Act Part C early intervention or state-funded services (*11*). 

Children met the surveillance ASD case definition if they were aged 4 years in 2018 (born in 2014), had a parent or guardian living in the surveillance area for at least one day during 2018, and had ever received a written ASD diagnostic statement (diagnosis) by a qualified professional, a special education classification of ASD (eligibility), or an ASD ICD-9 code between 299.00 and 299.99 or an ICD-10 code in the F84 range (*12*). Children with an ICD code for F84.2 (Rett syndrome) and no other indicators of ASD did not meet the ASD case definition. If none of these conditions were met but records contained a qualified professional’s statement indicating a suspicion of ASD, the children were classified as having suspected ASD. If the ASD or suspected ASD case definition was met, additional relevant information was collected from records, including demographics, comprehensive evaluations, and intelligence quotient (IQ) assessments.

### Additional Data Sources and Variable Definitions

The total number of children aged 4 years living in each surveillance area was obtained from the National Center for Health Statistics vintage 2019 postcensal bridged-race population estimates for 2018 (https://www.cdc.gov/nchs). For subcounty study areas in Arizona, California, Minnesota, and New Jersey, population estimates were standardized using public school enrollment counts (https://nces.ed.gov/ccd/files.asp) of school districts included in surveillance areas. Full details (Supplementary Methods, https://stacks.cdc.gov/view/cdc/111177) and denominators (Supplementary Table 1, https://stacks.cdc.gov/view/cdc/111177) are available.

Sites linked children’s records to state birth certificate data to supplement demographic information as needed. Children were classified as having co-occurring intellectual disability if they had a score ≤70 on their most recent IQ test or an examiner’s statement of intellectual disability in a developmental evaluation. Evaluation by age 36 months was calculated using the earliest recorded evaluation for each child.

As an indicator of socioeconomic status (SES), sites linked census-tract–level data about median household income (MHI) and population estimates from the 2018 American Community Survey (ACS) 5-year estimates (https://www2.census.gov/programs-surveys/acs) to records of children on the basis of their 2018 address. Census tracts for all sites combined were grouped into low, middle, and high MHI tertiles that included roughly equal populations of children aged 4 years (0–4-year age group population divided by five to estimate a single year of age) (Supplementary Table 2, https://stacks.cdc.gov/view/cdc/111177). Census-tract information to link to MHI was available for 94% of children aged 4 years; others met residency requirements on the basis of school attendance or receipt of services that required living in the surveillance area during 2018.

### Analytic Methods

ASD prevalence was calculated as the number of children who met the ASD surveillance case definition per 1,000 children aged 4 years living in the surveillance area. Prevalence was calculated overall, by sex, and by race and ethnicity for non-Hispanic White (White), non-Hispanic Black (Black), Hispanic, Asian/Pacific Islander (A/PI), and American Indian/Alaska Native (AI/AN) children. No children aged 4 years were lacking sex information; 245 children were of other (including multiracial) or unknown race and were excluded from analyses stratified by race and ethnicity. Case counts and denominators for estimates among children aged 4 years are available (Supplementary Table 1, https://stacks.cdc.gov/view/cdc/111177). If the prevalence estimate relative standard error was ≥30%, the estimate was considered unstable and suppressed. Prevalence ratios were used to compare prevalence by sex, race, and ethnicity; prevalence ratios using at least one unstable estimate were likewise suppressed.

Cumulative incidence of ASD diagnosis or eligibility per 1,000 children aged 4 years was calculated by dividing the total number of children with an ASD diagnosis or eligibility (age in months if an ICD code was not available) by each month of age by the population denominator for children aged 4 years or 8 years in 2018. Cumulative incidence of ASD diagnosis or eligibility per 1,000 children by MHI tertile used the appropriate ACS 5-year population denominator (0–4-year age group population divided by five to estimate a single year of age) for census tracts included in the tertile (Supplementary Table 2, https://stacks.cdc.gov/view/cdc/111177). Risk ratios were used to compare cumulative incidence of ASD diagnosis or eligibility by age 48 months between children aged 4 years and 8 years in 2018. Cochran-Armitage tests of trend were used to test trends of cumulative incidence of ASD diagnosis or eligibility by age 48 months across MHI tertiles among children aged 4 years in 2018.

The Wilson score method was used to calculate 95% confidence intervals (CIs) for prevalence, prevalence ratios, cumulative incidence, and risk ratios. Prevalence and risk ratios were considered significant at an alpha level of 0.05 when the 95% CIs did not include the null value of 1.0. The male-to-female prevalence ratio among children aged 4 years was compared between sites and with the ratio among children aged 8 years in the overall ADDM Network using the Mantel-Haenszel test of homogeneity (*11*). Chi-square tests were used to compare differences in distributions between groups for analyses of co-occurring intellectual disability, evaluation by age 36 months, directly comparing high to low MHI tertile, and having ASD versus suspected ASD. Cochran-Armitage tests of trend were used to assess trends across MHI tertiles. Mantel-Haenszel, chi-square, and Cochran-Armitage tests of trend were considered significant if the p value was <0.05. R software (version 4.0.3; R Foundation) was used for all data analysis and visualizations.

## Results

### Prevalence and Characteristics of Children Aged 4 Years with ASD

**FIGURE 1:**
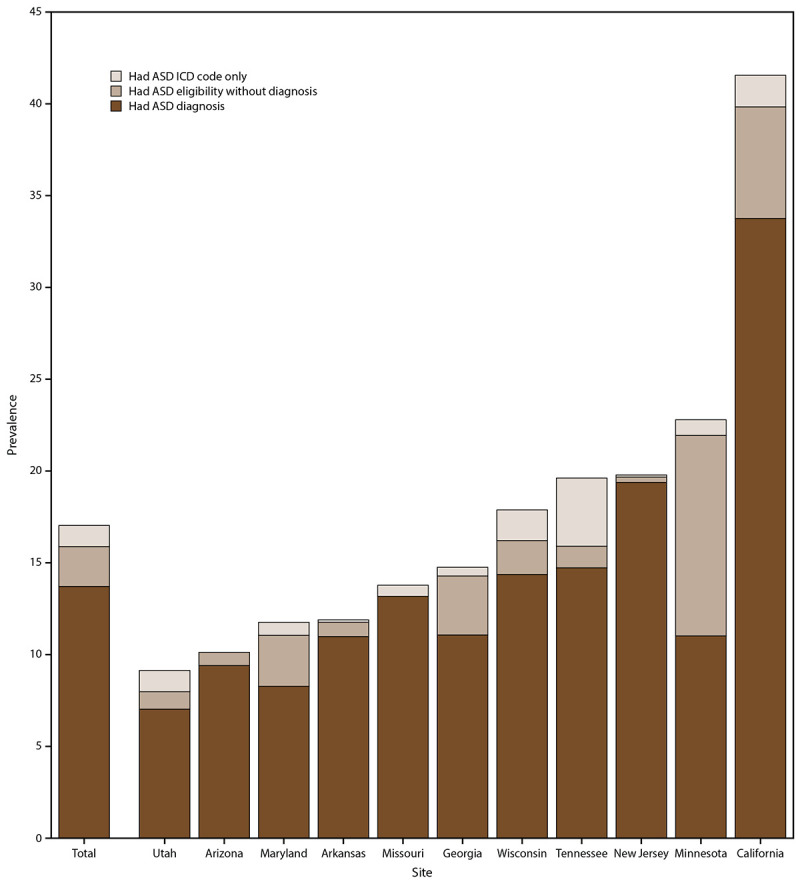
Prevalence of autism spectrum disorder per 1,000 children aged 4 years, by identification type and site — Autism and Developmental Disabilities Monitoring Network, 11 sites, United States, 2018 **Abbreviations:** ASD = autism spectrum disorder; ICD = International Classification of Diseases.

For 2018, ASD prevalence in the 11 sites combined was 17.0 per 1,000 (1 in 59) children aged 4 years. Estimates ranged widely across the sites, from 9.1 per 1,000 in Utah to 41.6 per 1,000 in California (Table 1). Overall, 80.5% of children who met the ADDM Network case definition had an ASD diagnosis (range: 48.3%–98.0%); 42.9% had ASD eligibility (range: 3.3%–88.3%); and 72% had an ASD ICD code (range: 44.0%–97.6%). Two or more components of the case definition were present in records for 74% of children aged 4 years (Supplementary Figure 1, https://stacks.cdc.gov/view/cdc/111177). Overall, 12.8% of children with ASD did not have a documented diagnosis but did have an ASD eligibility classification (with or without an ICD code), and 6.7% had an ICD code only (Figure 1).

#### Demographics

Among boys aged 4 years, prevalence was 25.9 per 1,000, which was 3.4 times as high as the 7.7 per 1,000 prevalence among girls aged 4 years (Table 2). No evidence of heterogeneity of the male-to-female ratio among children aged 4 years across sites was observed; however, the overall male-to-female ratio for children aged 4 years was lower than the 4.2 male-to-female ratio for children aged 8 years (*11*).

**TABLE 2:** Prevalence of autism spectrum disorder per 1,000 children aged 4 years, by sex — Autism and Developmental Disabilities Monitoring Network, 11 sites, United States, 2018

Site	Male ASD prevalence (95% CI)*	Female ASD prevalence (95% CI)*	Male-to-female prevalence ratio (95% CI)*^,†^
Arizona	16.6 (13.8–19.8)	3.5 (2.4–5.2)	4.7 (3.1–7.3)
Arkansas	19.2 (16.4–22.5)	4.1 (2.9–5.9)	4.6 (3.2–6.8)
California	63.6 (58.6–68.9)	18.2 (15.5–21.3)	3.5 (2.9–4.2)
Georgia	23.5 (20.9–26.5)	5.7 (4.5–7.3)	4.1 (3.1–5.4)
Maryland	18.2 (15.8–21.0)	5.0 (3.8–6.6)	3.7 (2.7–5.0)
Minnesota	34.8 (30.2–40.0)	10.6 (8.1–13.7)	3.3 (2.4–4.4)
Missouri	20.2 (17.9–22.8)	7.1 (5.7–8.8)	2.8 (2.2–3.6)
New Jersey	30.3 (26.9–34.1)	8.8 (7.0–11.0)	3.5 (2.7–4.5)
Tennessee	29.2 (26.4–32.2)	9.8 (8.2–11.7)	3.0 (2.4–3.7)
Utah	13.7 (11.8–15.9)	4.3 (3.3–5.7)	3.2 (2.3–4.3)
Wisconsin	26.2 (23.7–28.9)	9.0 (7.6–10.7)	2.9 (2.4–3.5)
**Total**	**25.9 (25.0–26.9)**	**7.7 (7.2–8.2)**	**3.4 (3.1–3.6)**

**Abbreviations:** ASD = autism spectrum disorder; CI = confidence interval.

* 95% CIs were calculated using the Wilson score method.

^†^ Prevalence significantly higher among males than females at all sites (prevalence ratio 95% CIs do not include 1.0).

Population distributions and ASD prevalence estimates by race and ethnicity varied among sites (Table 3) (Supplementary Table 1, https://stacks.cdc.gov/view/cdc/111177). Overall, A/PI children had the highest prevalence of ASD at 22.7 per 1,000 children aged 4 years, followed by Hispanic children at 21.1 per 1,000, Black children at 16.6 per 1,000, White children at 12.9 per 1,000, and AI/AN children at 11.5 per 1,000.

**TABLE 3:** Prevalence of autism spectrum disorder per 1,000 children aged 4 years, by race/ethnicity* — Autism and Developmental Disabilities Monitoring Network, 11 sites, 2018

Site	ASD prevalence (95% CI)^†^					Prevalence ratio (95% CI)					
	White, non-Hispanic	Black, non-Hispanic	Hispanic	Asian/Pacific Islander	American Indian/Alaska Native	White, non-Hispanic to Black, non-Hispanic	White, non-Hispanic to Hispanic	White, non-Hispanic to Asian/Pacific Islander	Black, non-Hispanic to Hispanic	Black, non-Hispanic to Asian/Pacific Islander	Hispanic to Asian/Pacific Islander
Arizona	13.2 (10.6–16.4)	—^§^	8.5 (6.5–11.3)	—^§^	—^§^	—^§^	1.6 (1.1–2.2)^¶^	—^§^	—^§^	—^§^	—^§^
Arkansas	11.4 (9.5–13.7)	10.5 (7.8–14.2)	—^§^	—^§^	—^§^	1.1 (0.8–1.5)	—^§^	—^§^	—^§^	—^§^	—^§^
California	26.0 (21.9–30.8)	30.6 (23.1–40.5)	45.5 (41.1–50.3)	41.7 (34.3–50.6)	—^§^	0.8 (0.6–1.2)	0.6 (0.5–0.7)^¶^	0.6 (0.5–0.8)^¶^	0.7 (0.5–0.9)^¶^	0.7 (0.5–1.0)	1.1 (0.9–1.4)
Georgia	12.0 (9.6–15.0)	17.1 (14.7–20.0)	10.0 (7.7–13.0)	17.7 (12.9–24.3)	—^§^	0.7 (0.5–0.9)^¶^	1.2 (0.9–1.7)	0.7 (0.5–1.0)	1.7 (1.3–2.3)^¶^	1.0 (0.7–1.4)	0.6 (0.4–0.9)^¶^
Maryland	9.1 (7.5–11.1)	13.5 (10.6–17.1)	8.8 (5.5–14.0)	15.5 (10.8–22.3)	—^§^	0.7 (0.5–0.9)^¶^	1.0 (0.6–1.7)	0.6 (0.4–0.9)^¶^	1.5 (0.9–2.6)	0.9 (0.6–1.3)	0.6 (0.3–1.0)
Minnesota	16.6 (13.6–20.3)	23.5 (18.4–30.0)	24.4 (17.0–34.8)	30.8 (21.3–44.5)	—^§^	0.7 (0.5–1.0)^¶^	0.7 (0.5–1.0)	0.5 (0.4–0.8)^¶^	1.0 (0.6–1.5)	0.8 (0.5–1.2)	0.8 (0.5–1.3)
Missouri	13.1 (11.4–14.9)	11.5 (9.2–14.5)	—^§^	30.0 (20.7–43.3)	—^§^	1.1 (0.9–1.5)	—^§^	0.4 (0.3–0.6)^¶^	—^§^	0.4 (0.3–0.6)^¶^	—^§^
New Jersey	15.2 (12.0–19.2)	20.0 (16.6–24.1)	20.6 (17.4–24.4)	16.4 (10.1–26.5)	—^§^	0.8 (0.6–1.0)	0.7 (0.6–1.0)^¶^	0.9 (0.5–1.6)	1.0 (0.8–1.2)	1.2 (0.7–2.0)	1.3 (0.8–2.1)
Tennessee	16.8 (14.9–18.9)	21.1 (17.4–25.5)	24.8 (20.1–30.5)	20.0 (12.5–31.8)	—^§^	0.8 (0.6–1.0)^¶^	0.7 (0.5–0.9)^¶^	0.8 (0.5–1.4)	0.9 (0.6–1.1)	1.1 (0.6–1.7)	1.2 (0.7–2.1)
Utah	7.7 (6.5–9.1)	—^§^	10.8 (8.3–13.9)	9.9 (5.7–17.2)	—^§^	—^§^	0.7 (0.5–1.0)^¶^	0.8 (0.4–1.4)	—^§^	—^§^	1.1 (0.6–2.0)
Wisconsin	12.7 (11.1–14.5)	18.1 (15.0–22.0)	30.4 (25.7–36.0)	21.9 (15.8–30.3)	—^§^	0.7 (0.6–0.9)^¶^	0.4 (0.3–0.5)^¶^	0.6 (0.4–0.8)^¶^	0.6 (0.5–0.8)^¶^	0.8 (0.6–1.2)	1.4 (1.0–2.0)
**Total**	**12.9 (12.3–13.6)**	**16.6 (15.4–17.8)**	**21.1 (19.8–22.4)**	**22.7 (20.3–25.4)**	**11.5 (7.2–18.4)**	**0.8 (0.7–0.9)^¶^**	**0.6 (0.6–0.7)^¶^**	**0.6 (0.5–0.6)^¶^**	**0.8 (0.7–0.9)^¶^**	**0.7 (0.6–0.8)^¶^**	**0.9 (0.8–1.1)**

**Abbreviations:** ASD = autism spectrum disorder; CI = confidence interval.

* Excludes children of other (including multiracial) or unknown race.

^†^ 95% CIs were calculated using the Wilson score method.

^§^ Estimate was suppressed because standard error for prevalence was ≥30% of estimate or prevalence ratio was based on an estimate that was suppressed.

^¶^ Significant prevalence ratio (unrounded 95% CIs do not include 1.0).

Overall, prevalence was lower among White children than among Black children (prevalence ratio: 0.8), Hispanic children (prevalence ratio: 0.6), and A/PI children (prevalence ratio: 0.6) (Table 3). The prevalence among Black children, although significantly higher than among White children, also was significantly lower than among Hispanic and A/PI children (prevalence ratios: 0.8 and 0.7, respectively). Prevalence was not different between Hispanic and A/PI children.

Sites typically matched the overall pattern of lower ASD prevalence among White children (Table 3). Prevalence among White children was lower than among Black, Hispanic, and A/PI children at five of 11 sites each. The two exceptions to the overall patterns were in Arizona, where prevalence among White children was 1.6 times as high as among Hispanic children, and in Georgia, where prevalence among Black children was 1.7 times as high as among Hispanic children.

#### Co-Occurring Intellectual Disability

Overall, 53.4% of children aged 4 years with ASD had information on intellectual ability available (range: 18.6% in Missouri to 77.7% in California) (Supplementary Table 3, https://stacks.cdc.gov/view/cdc/111177). Among the group of children with data available, 51.6% of children with ASD had co-occurring intellectual disability according to the surveillance case definition (range: 23.1% in California to 76.3% in Maryland) (Table 4). Intellectual disability case status as a proportion of ASD prevalence is available (Supplementary Figure 2, https://stacks.cdc.gov/view/cdc/111177).

**TABLE 4:** Presence of co-occurring intellectual disability among children aged 4 years with autism spectrum disorder and available intellectual disability information, by site and selected characteristics — Autism and Developmental Disabilities Monitoring Network, 11 sites, 2018

Site/Characteristic	No. with intellectual disability information	With co-occurring intellectual disability
		No. (%)*
**Site**		
Arizona	94	54 (57.4)
Arkansas	121	75 (62.0)
California	542	125 (23.1)
Georgia	192	115 (59.9)
Maryland	118	90 (76.3)
Minnesota	159	90 (56.6)
Missouri	63	31 (49.2)
New Jersey	205	114 (55.6)
Tennessee	241	162 (67.2)
Utah	90	53 (58.9)
Wisconsin	178	124 (69.7)
**Total**	**2,003**	**1,033 (51.6)**
**Sex**		
Female	434	230 (53.0)
Male	1,569	803 (51.2)
**Race/Ethnicity** ^†^		
White, non-Hispanic	744	371 (49.9)
Black, non-Hispanic	394	264 (67.0)
Asian/Pacific Islander	169	81 (47.9)
Hispanic	554	252 (45.5)
**Median household income tertile^§^**		
Low	685	411 (60.0)
Middle	679	359 (52.9)
High	631	256 (40.6)

* Chi-square p values for comparisons: male to female: p = 0.54; White to Asian/Pacific Islander: p = 0.71; White to Hispanic: p = 0.13; White to Black, Asian/Pacific Islander to Black, Hispanic to Black: p<0.01; Cochran-Armitage test for trend for median household income tertile: p<0.01.

^†^ Excludes children of other (including multiracial) or unknown race.

^§^ Limited to 3,534 children with median household income information (218 children were not able to be matched to census tract and two children were living in census tracts with suppressed median household income estimates).

The percentage of children with co-occurring intellectual disability was not different between boys (51%) and girls (53%) with ASD (Table 4). The percentage of children with ASD with intellectual disability was similar among White, A/PI, and Hispanic children (50%, 48%, and 45%, respectively) but was higher among Black children (67%). Co-occurring intellectual disability was associated with lower MHI; 41% of those in the high MHI tertile group had intellectual disability compared with 60% of those in the low MHI tertile group.

#### First Evaluation

Overall, 72% of children with ASD with an evaluation had their earliest recorded evaluation by age 36 months, with variability across sites ranging from 66% in Utah and Wisconsin to 83% in Maryland (Table 5). Evaluation by age 36 months was similar by sex and by racial and ethnic groups. The percentage of children with earliest recorded evaluation by age 36 months was similar for children classified as having intellectual disability (78%) or not (80%) but was lower among children with unknown intellectual disability case status (63%).

**TABLE 5:** Percentage of children aged 4 years with autism spectrum disorder who had earliest recorded evaluation by age 36 months, by site and selected characteristics — Autism and Developmental Disabilities Monitoring Network, 11 sites, United States, 2018

Site/Characteristic	No. with evaluation	Evaluated by age 36 mos
		No. (%)*
**Site**		
Arizona	141	115 (81.6)
Arkansas	182	124 (68.1)
California	679	504 (74.2)
Georgia	285	211 (74.0)
Maryland	183	152 (83.1)
Minnesota	228	156 (68.4)
Missouri	334	248 (74.3)
New Jersey	338	250 (74.0)
Tennessee	476	322 (67.6)
Utah	202	134 (66.3)
Wisconsin	473	314 (66.4)
**Total**	**3,521**	**2,530 (71.9)**
**Sex**		
Female	791	564 (71.3)
Male	2,730	1,966 (72.0)
**Race/Ethnicity^†^**		
White, non-Hispanic	1,393	1,003 (72.0)
Black, non-Hispanic	706	499 (70.7)
Asian/Pacific Islander	280	199 (71.1)
Hispanic	894	653 (73.0)
**Co-occurring intellectual disability**		
Intellectual disability	1,028	803 (78.1)
No intellectual disability	967	770 (79.6)
Unknown	1,526	957 (62.7)
**Median household income tertile** ^§^		
Low	1,274	911 (71.5)
Middle	1,165	860 (73.8)
High	999	750 (75.1)

* Chi-square p values for comparisons: sex: p = 0.73; race: p = 0.75; intellectual disability to no intellectual disability: p = 0.44; intellectual disability to unknown, no intellectual disability to unknown: p<0.01; low to high median household income: p = 0.06; Cochran-Armitage test for trend for median household income tertile: p = 0.45.

^†^ Excludes children of other (including multiracial) or unknown race.

^§^ Limited to 3,534 children with median household income information (218 children were not able to be matched to census tract, and two children were living in census tracts with suppressed median household income estimates).

No trend was observed in evaluation by age 36 months by MHI tertile overall. However, among children with intellectual disability, a lower percentage was evaluated by age 36 months among the low SES group (72%) than among the high SES group (84%) (Supplementary Table 4, https://stacks.cdc.gov/view/cdc/111177). In contrast, the percentages of children evaluated by age 36 months were similar across MHI tertiles among children without intellectual disability (Supplementary Table 4, https://stacks.cdc.gov/view/cdc/111177).

#### Cumulative Incidence of ASD Diagnosis or Eligibility Compared with Children Aged 8 Years

**FIGURE 2:**
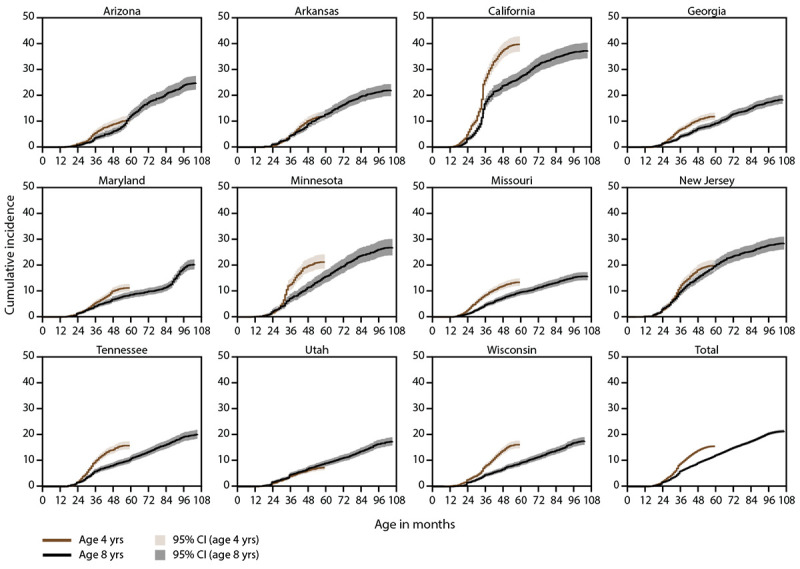
Cumulative incidence of autism spectrum disorder diagnosis or eligibility per 1,000 children aged 4 or 8 years, by site and month of age at identification — Autism and Developmental Disabilities Monitoring Network, 11 sites, United States, 2018 **Abbreviation:** CI = confidence interval.

Compared with children aged 8 years (born in 2010), children aged 4 years (born in 2014) had a higher cumulative incidence of ASD diagnosis or eligibility by age 48 months (Figure 2). At age 48 months, overall cumulative incidence was 13.6 per 1,000 children born in 2014 and 8.9 per 1,000 children born in 2010, meaning ASD diagnosis or eligibility was 1.5 times as high in the group born in 2014 (Supplementary Table 5, https://stacks.cdc.gov/view/cdc/111177). The pattern was evident for nine of 11 sites (range: 1.2 times as high in New Jersey to 2.1 times as high in Wisconsin); however, the cumulative incidence curve for children born in 2014 overlapped with the curve for children born in 2010 in Arkansas and Utah (Figure 2).

#### Cumulative Incidence of ASD Diagnosis or Eligibility by SES

Cumulative incidence of ASD diagnosis or eligibility by age 48 months was 13.8, 12.8, and 11.7 per 1,000 children aged 4 years among low, middle, and high MHI tertiles, respectively. An overall association of higher ASD identification by age 48 months with lower MHI was observed. Five of 11 sites (Arizona, California, Tennessee, Utah, and Wisconsin) demonstrated the same association of higher cumulative ASD incidence with lower MHI tertile (Table 6) (Supplementary Figure 3, https://stacks.cdc.gov/view/cdc/111177). Cumulative incidence by age 48 months was higher with higher MHI in one site (Missouri). Trends were not observed among the other five sites.

**TABLE 6:** Cumulative incidence of autism spectrum disorder diagnosis or eligibility by age 48 months per 1,000 children aged 4 years, by site and median household income tertile* — Autism and Developmental Disabilities Monitoring Network, 11 sites, United States, 2018

Site	Low MHI tertile	Middle MHI tertile	High MHI tertile
	Rate (95% CI)^†^	Rate (95% CI)^†^	Rate (95% CI)^†^
Arizona^§^	9.0 (7.0–11.6)	6.1 (4.4–8.6)	5.0 (3.4–7.4)
Arkansas	8.9 (7.2–11.0)	10.6 (8.1–14.0)	15.0 (9.1–24.6)
California^§^	36.3 (31.5–41.7)	27.6 (24.2–31.6)	26.1 (22.9–29.7)
Georgia	9.3 (7.5–11.5)	9.7 (7.7–12.2)	12.2 (9.8–15.1)
Maryland	7.4 (4.6–12.0)	8.3 (6.4–10.9)	7.8 (6.3–9.7)
Minnesota	18.1 (14.3–23.0)	15.8 (12.6–19.8)	14.6 (11.4–18.6)
Missouri^§^	9.4 (7.5–11.7)	12.3 (10.2–14.9)	13.2 (10.9–16.1)
New Jersey	17.3 (14.7–20.3)	19.9 (15.6–25.5)	18.3 (15.1–22.3)
Tennessee^§^	14.6 (12.3–17.2)	15.6 (13.3–18.2)	10.1 (7.8–13.0)
Utah^§^	11.9 (8.9–15.9)	5.9 (4.6–7.7)	4.4 (3.3–5.8)
Wisconsin^§^	14.2 (12.2–16.5)	12.3 (10.2–14.7)	9.3 (7.4–11.7)
**Total^§^**	**13.8 (13.0–14.6)**	**12.8 (12.1–13.7)**	**11.7 (11.0–12.5)**

**Abbreviations:** CI = confidence interval; MHI = median household income.

* Limited to 3,534 children with MHI information (218 children were not able to be matched to census tract and two children were living in census tracts with suppressed MHI estimates).

^†^ 95% CIs were calculated using the Wilson score method.

^§^ Cochran-Armitage test for trend p<0.05.

### Prevalence and Characteristics of Children Aged 4 Years with Suspected ASD

The overall prevalence of children without an ASD diagnosis, eligibility, or ICD code but who were suspected by a qualified professional of having ASD was 2.6 per 1,000 children aged 4 years (range: 0.4 per 1,000 in California to 4.9 per 1,000 in Arizona) (Table 7) (Supplementary Figure 4, https://stacks.cdc.gov/view/cdc/111177). Across sites, six children met the ASD case definition for every child meeting the suspected case definition. The ratio of children with ASD to children with suspected ASD ranged from 116:1 in California to 2:1 in Arizona. The combined prevalence of ASD and suspected ASD (19.7 per 1,000 children aged 4 years) was still lower than ASD prevalence observed among children aged 8 years (23.0 per 1,000 children aged 8 years) (*11*).

**TABLE 7:** Prevalence of suspected autism spectrum disorder per 1,000 children aged 4 years — Autism and Developmental Disabilities Monitoring Network, 11 sites, United States, 2018

Site	No. of children aged 4 yrs with suspected ASD	Suspected ASD prevalence rate (95% CI)*	Ratio of ASD prevalence to suspected ASD prevalence
Arizona	68	4.9 (3.9–6.2)	2:1
Arkansas	72	4.7 (3.7–5.9)	3:1
California	6	0.4 (0.2–0.8)	116:1
Georgia	69	3.0 (2.4–3.8)	5:1
Maryland	48	2.4 (1.8–3.2)	5:1
Minnesota	23	2.2 (1.5–3.3)	10:1
Missouri	36	1.5 (1.1–2.0)	9:1
New Jersey	37	2.1 (1.6–2.9)	9:1
Tennessee	33	1.3 (0.9–1.8)	15:1
Utah	86	3.4 (2.8–4.2)	3:1
Wisconsin	102	3.6 (2.9–4.3)	5:1
**Total**	**580**	**2.6 (2.4–2.9)**	**6:1**

**Abbreviations:** ASD = autism spectrum disorder; CI = confidence interval.

* 95% CIs were calculated using the Wilson score method.

Children with ASD and children with suspected ASD did not differ by sex (78% male and 76% male, respectively). Differences were observed by race and ethnicity, with more White children (50%) and fewer A/PI children (5%) in the suspected ASD group than in the ASD group (43% White; 9% A/PI) (Table 8). In addition, among children with data available, co-occurring intellectual disability was less common among children with suspected ASD (41%) than among those with ASD (52%). Similar proportions of children with ASD or suspected ASD were evaluated by age 36 months. Although ASD was identified more frequently among children in lower SES groups, no association was observed between suspected ASD and SES.

**TABLE 8:** Characteristics of children aged 4 years with autism spectrum disorder and with suspected autism spectrum disorder — Autism and Developmental Disabilities Monitoring Network, 11 sites, United States, 2018

Characteristic	Children with ASD	Children with suspected ASD*
	No. (%)	No. (%)
**Sex**		
Female	830 (22.1)	140 (24.1)
Male	2,924 (77.9)	440 (75.9)
**Race/Ethnicity^†^**		
White, non-Hispanic	1,487 (42.6)	272 (49.5)
Black, non-Hispanic	771 (22.1)	127 (23.1)
Asian/Pacific Islander	302 (8.6)	25 (4.6)
Hispanic	932 (26.7)	125 (22.8)
**Intellectual disability^§^**		
Intellectual disability	1,033 (51.6)	106 (41.4)
**Evaluation** ^¶^		
Earliest recorded evaluation by age ≤36 mos	2,530 (71.9)	401 (69.1)
Median household income tertile**		
Low	1,309 (37.0)	183 (32.3)
Middle	1,199 (33.9)	204 (36.0)
High	1,026 (29.0)	179 (31.6)

**Abbreviation:** ASD = autism spectrum disorder.

* Chi-square p values for comparisons: sex: p = 0.30; race: p<0.01; intellectual disability: p<0.01; evaluation: p = 0.61; Chi-square test of distribution of median household income tertile among children with ASD: p<0.01; Chi-square test of distribution of median household income tertile among children with suspected ASD: p = 0.38.

^†^ Excludes children of other (including multiracial) or unknown race.

^§^ Limited to children with intellectual disability data available.

^¶^ Limited to children with an evaluation (N = 3,521 children with ASD; N = 580 children with suspected ASD).

** Limited to 3,534 children with median household income information (218 children were not able to be matched to census tract and two children were living in census tracts with suppressed median household income estimates).

## Discussion

The ASD prevalence of 17.0 per 1,000 children aged 4 years for 2018 was higher than for 2016, when prevalence was 15.6 per 1,000 (*8*). The 2018 estimate for children aged 4 years was lower than the 23.0 per 1,000 ASD prevalence among children aged 8 years (*11*), a finding consistent with previous reports (*8*,*13*). When cumulative incidence of ASD diagnosis or eligibility by age 48 months was directly compared between children aged 4 years (born in 2014) and aged 8 years (born in 2010), early identification was higher in the 2014 cohort. This could suggest greater awareness of ASD among families, health care providers, and educators or greater emphasis on or capacity for evaluating and identifying children early in the more recent cohort.

Prevalence of ASD was higher than prevalence of suspected ASD, although the magnitude varied across sites. Adding the children identified with ASD to those suspected of having ASD at age 4 years is still less than the prevalence of ASD by age 8 years at all sites except California (*11*). This could suggest that many children who are not identified until after age 4 years also do not have a documented suspicion of ASD by age 4 years. Possible reasons are because developmental screening, referral, and evaluation is a multistep process with potential barriers at each level or because ASD might not be recognized until increasing social demands make a child’s symptoms more apparent (*14*). Improving awareness of early symptoms of developmental problems could prompt quicker action by parents, primary care providers, or educators. This is the goal of CDC’s “Learn the Signs. Act Early.” program, which provides free informational materials in multiple languages and a milestone tracker app (https://www.cdc.gov/ncbddd/actearly).

The site in California had the highest cumulative incidence of ASD by age 48 months and the fewest cases of suspected ASD and was the only site where prevalence among children aged 4 years was higher than among children aged 8 years (*11*). Multiple factors could contribute to ASD identification at this site. One of the site’s data sources was a state-funded regional center that serves persons with developmental disabilities and their families, including providing evaluations and service coordination (*15*). In addition, efforts have been made to promote early identification in the area. For example, the “Get SET Early” program has trained hundreds of pediatricians to developmentally screen children at well-baby examinations and refer them for evaluation when indicated (*16*). The contribution of these and other factors to variability in ASD identification across sites and over time merits further investigation.

Signs exist of changing patterns in identification by race and SES. In surveillance year 2018, ASD prevalence among Black children was higher than among White children at five of 11 sites and overall, the opposite of the pattern before surveillance year 2016 among children aged 8 years (*9*,*17*). In 2016, prevalence was higher among Hispanic compared with White children aged 4 years higher at one site (New Jersey) and overall (*8*). In 2018, prevalence was higher among Hispanic children than among White children at five sites, including New Jersey, and overall. Incidence of ASD identification was lower among Hispanic than among White children at only one site (Arizona). Comparisons with previous reports among children aged 4 years were limited by small numbers among non-White groups in surveillance year 2010 and a lack of aggregate estimates for surveillance years 2012 and 2014 (*13*). A change to the ASD case definition for 2018 that no longer required clinician review of records (*12*) could also contribute to higher prevalence among Black and Hispanic children who were less likely to have detailed health information available for review and therefore less likely to meet the case definition with previous methods (*18*).

With the expansion of ASD surveillance among children aged 4 years to the same surveillance areas as among children aged 8 years, surveillance year 2018 was the first year that sufficient data for A/PI and AI/AN children were available for presentation. The population of AI/AN children was small relative to other groups and thus could not be further stratified for comparisons. Targeted study is needed to provide information about characteristics of and potential service needs for AI/AN children with ASD.

An association of higher ASD prevalence and higher SES has been reported previously in ADDM Network data from surveillance years 2002–2010 (*19*). In 2018, the inverse association of higher ASD identification in the group with lower MIH was observed among children aged 4 years in the ADDM Network, consistent with reports using recent California Department of Developmental Services data (*20*) and England Spring School Census data (*21*). Children in the lower MHI group were more likely to have co-occurring intellectual disability, consistent with previous ADDM Network reports (*19*,*22*) and a study in France (*23*). A smaller percentage of children in the low MHI group with co-occurring intellectual disability were evaluated by age 36 months than in the high MHI group.

Children with co-occurring intellectual disability are more likely to be identified earlier than children without intellectual disability, as indicated by an earlier median age at identification among this group (*11*) as well as the consistently higher proportion of children with intellectual disability at age 4 years (*8*,*13*) than at age 8 years (*9*,*11*,*17*). Patterns observed among children at age 4 years could change by age 8 years if characteristics of children identified later (more likely without intellectual disability) are different. The sex ratio is one example of how differences in identification of children without intellectual disability can affect patterns observed at older ages, with a higher ratio of boys to girls at age 8 years than at age 4 years, consistent with previous reports (*8*,*9*).

Consistent with findings among children aged 8 years (*11*), a higher proportion of Black children aged 4 years with ASD have co-occurring intellectual disability. Underascertainment of Black children without intellectual disability across age groups, which has been reported among Black and Hispanic children relative to White children without intellectual disability from surveillance years 2002–2016 (*10*), could have a role in this disparity. Emphasis on equitable screening and provision of services is needed. Measuring screening and service access among groups would provide important context to these disparities and provide information for public health action.

## Limitations

The findings in this report are subject to at least five limitations. First, surveillance area populations are subsets of state populations and not necessarily generalizable to the entire state or United States. Second, sample size, demographics, and practices and policies vary among sites and could contribute to variability in findings. Third, differences in sites or study areas complicate comparisons over time. One way this limitation has been addressed is by assessing ASD identification among children aged 4 years and children aged 8 years in a single area in a single year so that these groups are directly comparable. Fourth, data completeness varied across sites (either because of lack of data sharing agreements, pandemic-related disruption, or incomplete information), which could result in incomplete or biased ascertainment of ASD or other characteristics such as intellectual disability. Finally, the surveillance case definition of intellectual disability is not the same as a clinical diagnosis of intellectual disability; IQ measurements might lack stability and children might not receive a diagnosis of intellectual disability at younger ages.

## Future Directions

The ADDM Network collects data about children evaluated for and identified with ASD. Adding data about screening and referrals could help identify any disparities that are barriers to timely and equitable ASD identification in communities. Increasing the number of sites with access to Individuals with Disabilities Education Act Part C early intervention data would enhance the ADDM Network’s ability to track early receipt of federally funded services. In addition, targeted collection of data, such as among AI/AN children, could provide much-needed information for underrepresented populations.

## Conclusion

Findings from the ADDM Network 2018 surveillance year indicate patterns of ASD identification among children aged 4 years that are different from what has been previously reported. ASD prevalence among children aged 4 years was higher among Black, Hispanic, and A/PI children than among White children, and higher cumulative incidence of ASD diagnosis or eligibility by age 48 months was associated with lower census-tract–level MHI. These findings could indicate improvements in ASD identification among historically underserved populations. Comparing across ADDM cohorts, cumulative incidence of ASD identification by age 48 months was higher among children born in 2014 compared with those born in 2010, which further suggests improvements in early identification have occurred. However, a lower percentage of children with ASD and intellectual disability in the low MHI tertile were evaluated by age 36 months than in the high MHI group, suggesting there could still be disparities in receiving timely evaluation. ASD prevalence among children aged 4 years was lower compared with children aged 8 years at most ADDM sites, even when prevalence of children with suspected ASD by age 4 years is included. This suggests many children with ASD identified by age 8 years do not have suspected ASD documented by age 4 years. Understanding factors that improve equitable access to early ASD identification and services could improve outcomes for children with ASD.
